# Exposure to *Porphyromonas gingivalis* Induces Production of Proinflammatory Cytokine via TLR2 from Human Respiratory Epithelial Cells

**DOI:** 10.3390/jcm9113433

**Published:** 2020-10-26

**Authors:** Norihisa Watanabe, Sho Yokoe, Yorimasa Ogata, Shuichi Sato, Kenichi Imai

**Affiliations:** 1 Department of Periodontology, Nihon University School of Dentistry, Chiyoda-ku, Tokyo 101-8310, Japan; watanabe.norihisa@nihon-u.ac.jp (N.W.); desh18040@g.nihon-u.ac.jp (S.Y.); satou.shuuichi@nihon-u.ac.jp (S.S.); 2 Department of Microbiology, Nihon University School of Dentistry, 1-8-13 Kanda-Surugadai, Chiyoda-ku, Tokyo 101-8310, Japan; 3 Department of Periodontology, Nihon University School of Dentistry at Matsudo, Chiba 271-8587, Japan; ogata.yorimasa@nihon-u.ac.jp

**Keywords:** aspiration pneumonia, chronic periodontitis, *Porphyromonas gingivalis*, proinflammatory cytokines, TLR2

## Abstract

Aspiration pneumonia is a major health problem owing to its high mortality rate in elderly people. The secretion of proinflammatory cytokines such as interleukin (IL)-8 and IL-6 by respiratory epithelial cells, which is induced by infection of respiratory bacteria such as *Streptococcus pneumoniae*, contributes to the onset of pneumonia. These cytokines thus play a key role in orchestrating inflammatory responses in the lower respiratory tract. In contrast, chronic periodontitis, a chronic inflammatory disease caused by the infection of periodontopathic bacteria, typically *Porphyromonas gingivalis*, is one of the most prevalent microbial diseases affecting humans globally. Although emerging evidence has revealed an association between aspiration pneumonia and chronic periodontitis, a causal relationship between periodontopathic bacteria and the onset of aspiration pneumonia has not been established. Most periodontopathic bacteria are anaerobic and are therefore unlikely to survive in the lower respiratory organs of humans. Therefore, in this study, we examined whether simple contact by heat-inactivated *P. gingivalis* induced proinflammatory cytokine production by several human respiratory epithelial cell lines. We found that *P. gingivalis* induced strong IL-8 and IL-6 secretion by BEAS-2B bronchial epithelial cells. *P. gingivalis* also induced strong IL-8 secretion by Detroit 562 pharyngeal epithelial cells but not by A549 alveolar epithelial cells. Additionally, Toll-like receptor (TLR) 2 but not TLR4 was involved in the *P. gingivalis*-induced proinflammatory cytokine production. Furthermore, *P. gingivalis* induced considerably higher IL-8 and IL-6 production than heat-inactivated *S. pneumoniae*. Our results suggest that *P. gingivalis* is a powerful inflammatory stimulant for human bronchial and pharyngeal epithelial cells and can stimulate TLR2-mediated cytokine production, thereby potentially contributing to the onset of aspiration pneumonia.

## 1. Introduction

Aspiration pneumonia is a major health problem because of its high mortality rates in elderly people who are frequently immunocompromised [1]. This occurs because of inflammation in the lower respiratory tract that is stimulated by mis-swallowed saliva entering through the pharynx [1]. Pneumonia is characterized by increased number of infiltrated inflammatory cells such as neutrophils and elevated levels of proinflammatory cytokines, including chemokines, in the lower respiratory tract [2,3]. The so-called respiratory bacteria, such as *Streptococcus pneumoniae*, are exogenous; hence, they initially infect lower respiratory epithelia and subsequently induce proinflammatory cytokine production [3]. In turn, secreted cytokines such as interleukin (IL)-8 recruit neutrophils to infected tissues, and IL-6 exerts proinflammatory effects on respiratory epithelial cells [3,4,5,6,7]. These cytokines thus play a key role in orchestrating inflammatory responses in the lower respiratory tract in the development of pneumonia.

Chronic periodontitis is defined as an endogenous microflora-associated chronic inflammatory disease that develops as a result of poor oral hygiene [8]. The disease which causes the destruction of the periodontium, including alveolar bone, is one of the most prevalent infectious diseases worldwide [8]. Chronic periodontitis is caused by increased inflammatory responses, including proinflammatory cytokine production executed by host local immunity that is triggered by and amplified in relation to an increase in the number of periodontopathic bacteria [8]. Among them, the most pathogenic bacterium is *Porphyromonas gingivalis*, a Gram-negative black-pigmented anaerobe that predominantly colonizes periodontal pockets and is detected on the tongue dorsum and in the saliva of patients with advanced chronic periodontitis [9]. *P. gingivalis* exerts its virulence by interacting with several host pattern recognition receptors such as Toll-like receptor (TLR) 2 and TLR4 [10].

Accumulating evidence indicates that chronic periodontitis is a risk factor for several systemic diseases such as pre-term birth, heart diseases, diabetes, and atherosclerosis [8]. In this regard, we have reported that a major metabolite of *P. gingivalis* possibly induces the reactivation of latently infected viruses, namely human immunodeficiency virus and Epstein–Barr virus [11,12]. Over the last two decades, chronic periodontitis has also been identified as a risk factor for aspiration pneumonia in the elderly [13,14]. *P. gingivalis* is isolated from bronchoalveolar lavage fluid (BALF) or sputum from patients with pneumonia [15,16,17,18]. In fact, an increase in teeth with periodontal pockets in the elderly is associated with increased mortality from aspiration pneumonia [19]. Furthermore, periodontal interventions such as oral hygiene instruction reduce the occurrence of aspiration pneumonia, even among high-risk individuals [20]. Further, in the elderly, there is an increased risk of aspiration because these individuals have reduced laryngopharyngeal sensitivity [21]. Thus, these observations suggest that periodontopathic bacteria present in saliva are aspirated through the pharynx into the lower respiratory tract, thereby contributing to the onset of aspiration pneumonia. Despite its importance, a causal relationship between *P. gingivalis* and aspiration pneumonia remains unexamined.

Based on the aforementioned observations, we have hypothesized that increased number of *P. gingivalis* in the aspiration may induce proinflammatory cytokine production by human respiratory epithelia. However, *P. gingivalis is* anaerobic, and it is therefore unlikely to exhibit stable virulence in the respiratory tract. In addition, the duration of *P. gingivalis* survival in vitro and in vivo remains unclear. Therefore, in this study, we used heat-inactivated bacterial cells to examine whether *P. gingivalis* induced the production of proinflammatory cytokines such as IL-8 and IL-6 by human bronchial, alveolar, and pharyngeal epithelial cells via TLR2 or TLR4. To the best of our knowledge, we have delineated for the first time a putative mechanism by which *P. gingivalis* induces inflammation in human respiratory epithelia, thereby potentially contributing to the onset of aspiration pneumonia.

## 2. Materials and Methods

### 2.1. Cell Culture and Reagents

Human bronchial (BEAS-2B), pharyngeal (Detroit 562), and alveolar (A549) epithelial cells were purchased from ATCC (Manassas, VA, USA) and maintained at 37 °C in Dulbecco’s modified Eagle’s medium (Sigma, St. Louis, MO, USA) containing 10% heat-inactivated fetal bovine serum (Thermo Fisher Scientific, Rockford, IL, USA), penicillin (100 U/mL), and streptomycin (100 μg/mL), as described previously. HEK293 human embryonic kidney cells stably transfected with an expression plasmid for either human TLR2 (293-TLR2; InvivoGen, San Diego, CA, USA) or human TLR4 (293-TLR4; InvivoGen) were purchased and maintained at 37 °C in Dulbecco’s modified Eagle’s medium (Sigma) containing 10% heat-inactivated fetal bovine serum (Thermo Fisher Scientific) in the presence of the antibiotic blasticidin (10 μg/mL) to maintain selection for these transfectants. Neutralizing antibodies against human TLR2 and TLR4 were obtained from R&D Systems (Minneapolis, MN, USA). Lipoteichoic acid (LTA) derived from *Staphylococcus aureus* as a TLR2 ligand and lipopolysaccharide (LPS) derived from *Escherichia coli* as a TLR4 ligand were also purchased from Sigma.

### 2.2. Bacterial Culture and Sample Adjustment

*P. gingivalis* ATCC 33,277 was cultured in brain heart infusion broth (BHIB: Becton, Dickinson and Company, Sparks, MD, USA) supplemented with 5 μg/mL hemin and 0.5 μg/mL menadione. *S. pneumoniae* ATCC 6303 was cultured in BHIB. The cultures were incubated at 37 °C for 24–72 h and grown in an anaerobic chamber (Te-Her Anaerobox, Hirasawa Co. Ltd., Tokyo, Japan) under an anaerobic condition of 10% H_2_, 10% CO_2_, and 80% N_2_. The bacterial cell density was adjusted to 1.0 × 10^10^ CFU/mL, and the bacterial suspension was heat-inactivated at 60 °C for 1 h and stored at −80 °C until use.

### 2.3. mRNA Preparation and Real-Time Polymerase Chain Reaction (PCR)

The experimental procedures for RNA purification and real-time PCR were performed as previously described [22]. Briefly, the cells were washed once with ice-cold phosphate-buffered saline and homogenized using QIAshredder (QIAGEN, Alameda, CA, USA), and total RNA was purified using an RNeasy Mini Kit (QIAGEN). For cDNA synthesis, total RNA (1 μg) was reverse-transcribed using an RNA PCR kit (PrimeScript; Takara Bio, Shiga, Japan). The resulting cDNA mixture was subjected to real-time PCR analysis using Premix Ex *Taq* solution (Takara Bio) containing 5 μM sense and antisense primers. The primer sequences used for the amplification of each gene were as follows: IL-8, forward (5′-CTT GTC ATT GCC AGC TGT GT-3′) and reverse (5′-TGA CTG TGG AGT TTT GGC TG-3′); IL-6, forward (5′-TTC GGT CCA GTT GCC TTC TC-3′) and reverse (5′-GAG GTG AGT GGC TGT CTG TG-3′); and glyceraldehyde-3-phosphate dehydrogenase (GAPDH), forward (5′-ACC AGC CCC AGC AAG AGC ACA AG-3′) and reverse (5′-TTC AAG GGG TCT ACA TGG CAA CTG-3′). PCR assays were performed using a TP-800 Thermal Cycler Dice Real-Time System (Takara Bio) and analyzed using the software provided by the device manufacturer. The thermal cycling conditions were 40 cycles of 95 °C for 5 s, 60 °C for 30 s, and 72 °C for 1 min. All real-time PCR experiments were performed in triplicate, and the specificity of each product was verified via melting curve analysis. Calculated gene expression levels were normalized to GAPDH mRNA levels.

### 2.4. Cytokine Measurements

IL-8 and IL-6 concentrations in the cell culture supernatants were measured using an enzyme-linked immunosorbent assay (ELISA) kit (R&D Systems) according to the manufacturer’s recommendations. All experiments were performed in triplicate, and data are presented as the mean ± SD.

### 2.5. Transfection and Luciferase Assay

For NF-κB reporter assays, HEK293, 293-TLR2, or 293-TLR4 cells were plated in 12-well plates (4 × 10^5^ cells/mL) and grown overnight. These cells were transfected with reporter plasmids using a Lipofectamine 2000 transfection reagent (Thermo Fisher Scientific) according to the manufacturer’s instructions. Further, 200 ng of 5xκB-luc, a plasmid in which luciferase gene expression is under the control of NF-κB, and 10 ng of an internal control plasmid pRL-TK, which expresses *Renilla reniformis* luciferase under the control of the TK promoter, were used for each transfection. Twenty-four hours after transfection, the cells were incubated in the presence of heat-inactivated *P. gingivalis*, LTA, and LPS for 24 h. Cells were harvested using Passive Lysis Buffer (Promega, Madison, WI, USA), and the extracts were assessed for luciferase activity using a Dual-Luciferase Assay System (Promega) as described previously [12]. Luciferase activity was normalized to *R. reniformis* luciferase activity, which acted as an as an internal control for transfection efficiency.

## 3. Results

### 3.1. P. gingivalis Induced IL-8 and IL-6 mRNA Expression and Protein Production by Human Bronchial Epithelial Cells

*P. gingivalis* is considered to orchestrate dysbiosis of periodontal flora, which triggers inflammatory responses including cytokine production in the periodontium and in turn causes alveolar bone-destruction as a major symptom of chronic periodontitis [8]. We therefore first examined the mRNA induction of IL-8 and IL-6 by BEAS-2B bronchial epithelial cells and pertinent protein release using real-time PCR and ELISA, respectively.

**Figure 1:**
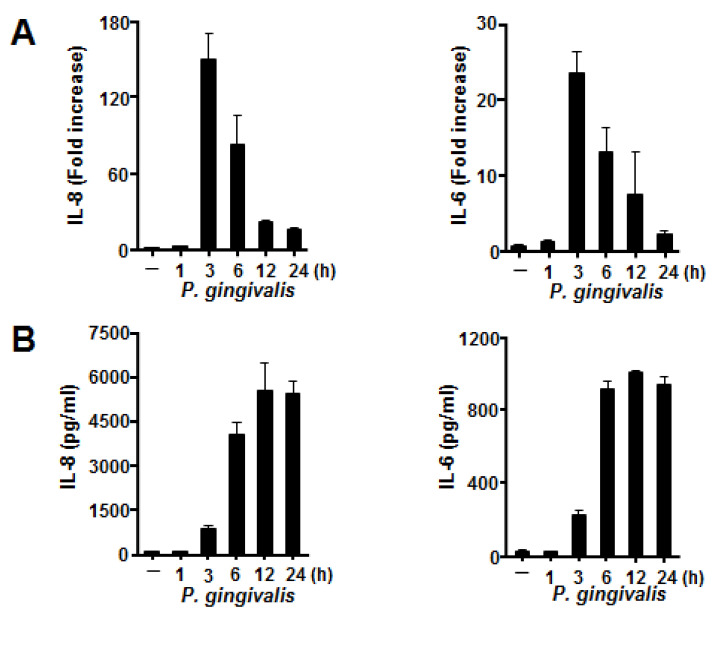

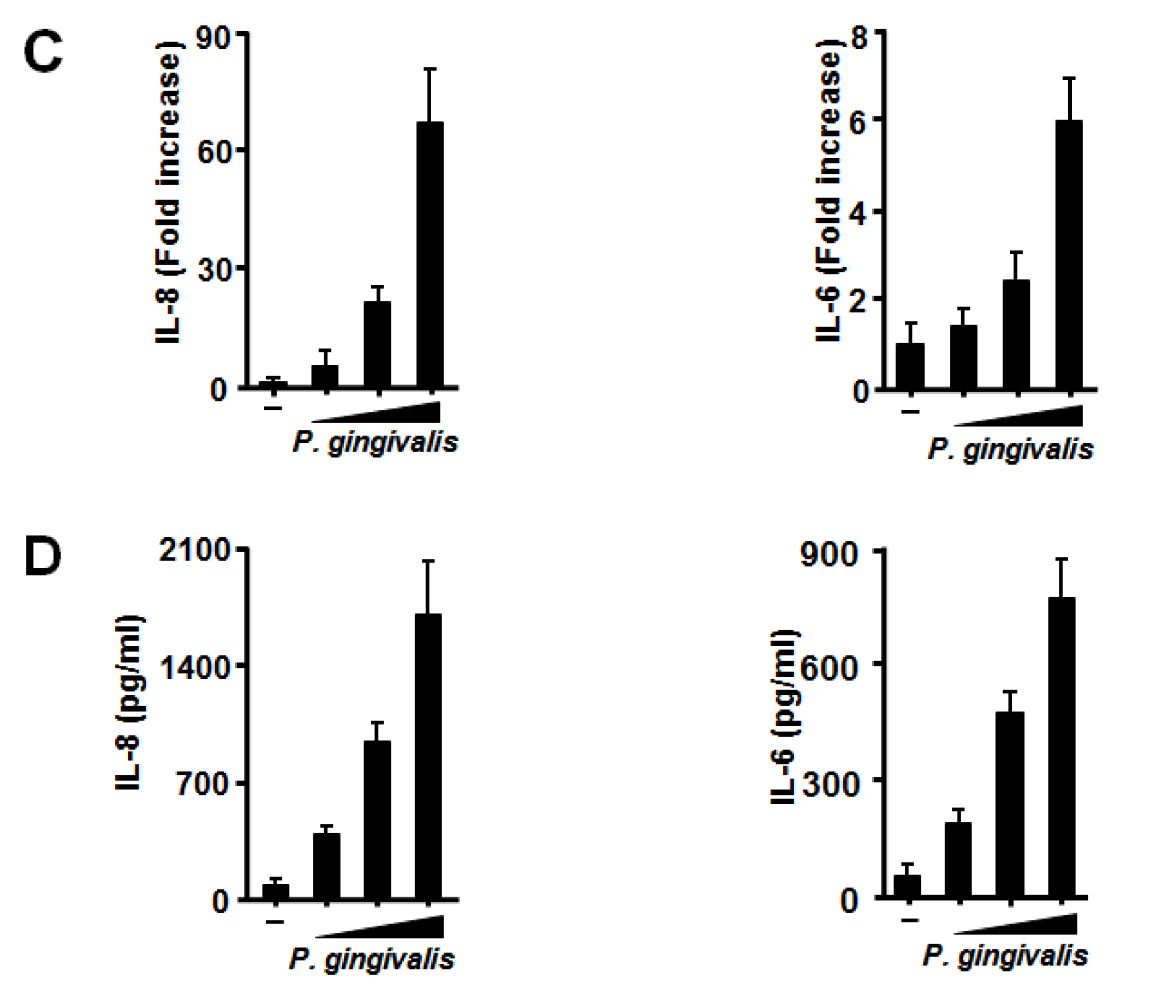
*Porphyromonas gingivalis*-induced mRNA expression and protein production of proinflammatory cytokines by human bronchial epithelial cells. BEAS-2B cells were exposed to heat-inactivated *P. gingivalis* (1 × 10^8^ CFU/mL) for the indicated times (**A**,**B**) and at different bacterial cell densities (1 × 10^7^, 0.5 × 10^8^, or 1 × 10^8^ CFU/mL) for 3 h (**C**) or 12 h (**D**). The cells were harvested, and IL-8 and IL-6 mRNA levels were measured using real-time polymerase chain reaction with specific primers. The mRNA levels were normalized to the GAPDH mRNA level and expressed as fold increases. IL-8 and IL-6 protein levels were determined by enzyme-linked immunosorbent assay and expressed as pg/mL. These experiments were performed in triplicate, and data are presented as the mean ± SD.

As shown in Figure 1A, *P. gingivalis* markedly upregulated IL-8 and IL-6 mRNA expression in BEAS-2B cells. The levels of both mRNAs rose after a 1 h exposure to *P. gingivalis* and peaked at 3 h of incubation (151 ± 21- and 24 ± 3- fold, respectively). As shown in Figure 1B, *P. gingivalis* elicited the release of both cytokines between 1 and 12 h in a time-dependent manner. To clarify the cause-and-effect relationship between the density of bacteria and the extent of cytokine mRNA expression and protein production levels, we used *P. gingivalis* at densities equivalent to 1 × 10^7^–1 × 10^8^ CFU/mL. As shown in Figure 1C,D, *P. gingivalis* induced cytokine production as well as mRNA expression in a density-dependent manner.

### 3.2. P. gingivalis Induced IL-8 and IL-6 Production by Human Pharyngeal Epithelial Cells but not by Human Alveolar Epithelial Cells

**Figure 2:**
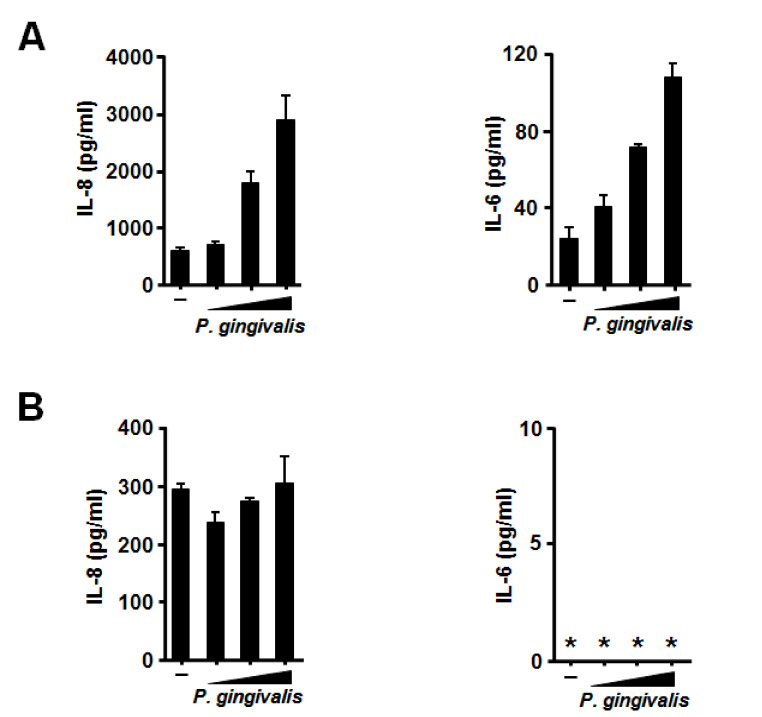
Effects of *Porphyromonas gingivalis* on interleukin (IL)-8 and IL-6 production by two human respiratory epithelial cells. Human pharyngeal (Detroit 562) (**A**) or alveolar (A549) (**B**) epithelial cells were incubated with heat-inactivated *P. gingivalis* (1 × 10^8^ CFU/mL) for 12 h. IL-8 and IL-6 protein levels in the cell culture supernatants were determin ed by enzyme-linked immunosorbent assay. These experiments were performed in triplicate, and data are presented as the mean ± SD. *; below the detection limit.

Because *P. gingivalis*-induced IL-8 and IL-6 production by BEAS-2B bronchial epithelial cells was observed, we examined whether pharyngeal epithelial cell line was also reactive to the stimulation with *P. gingivalis* because every material to be swallowed is retained in the pharynx for a while. As expected, stronger IL-8 production and much weaker IL-6 production than those by BEAS-2B cells were observed in Detroit 562 pharyngeal epithelial cells, which was in a density-dependent manner (Figure 2A). In contrast, proinflammatory cytokine production by *P. gingivalis* was not observed in A549 alveolar epithelial cells (Figure 2B).

### 3.3. P. gingivalis More Strongly Induced IL-8 and IL-6 Production than S. pneumoniae by Human Bronchial Epithelial Cells

**Figure 3:**
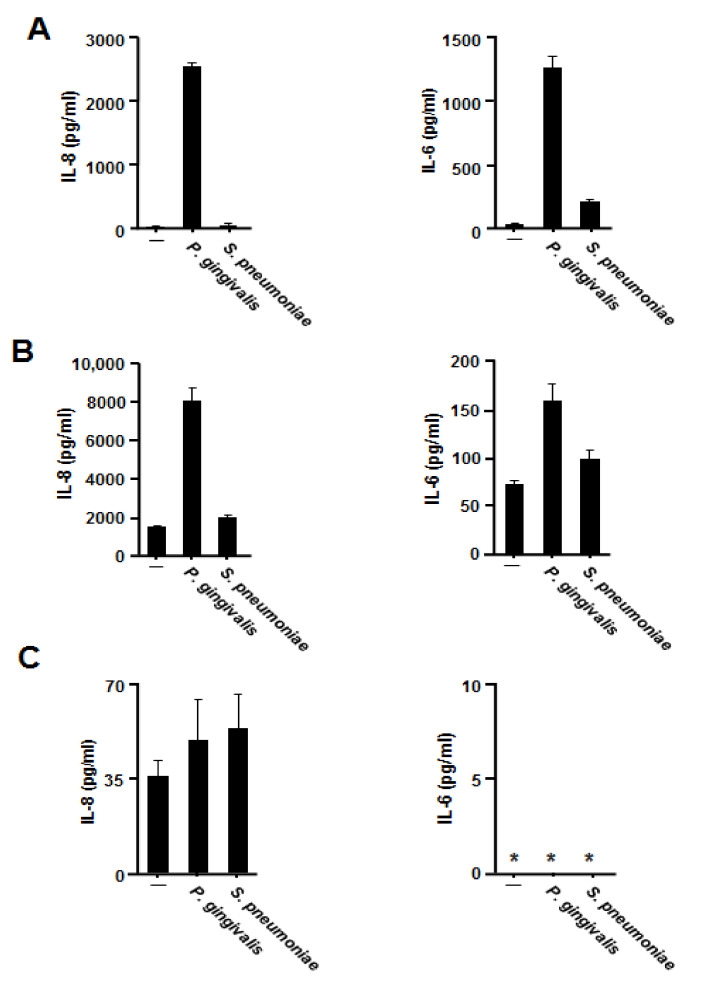
Comparison between the induction of proinflammatory cytokines by *Porphyromonas gingivalis* and by *Streptococcus pneumoniae*. BEAS-2B (**A**), Detroit 562 (**B**) or A549 (**C**) cells were incubated in the presence of heat-inactivated cells (1 × 10^8^ CFU/mL) of *P. gingivalis* or *S. pneumoniae* for 12 h. IL-8 and IL-6 levels in the culture supernatants were then determined using enzyme-linked immunosorbent assay. These experiments were performed in triplicate, and data are presented as the mean ± SD. *; below the detection limit.

*S. pneumoniae* contributes to the onset of aspiration pneumonia by inducing proinflammatory cytokines release. We therefore compared the inducibility of heat-inactivated bacterial cells between *P. gingivalis* and *S. pneumoniae* by human bronchial and pharyngeal epithelial cells. As shown in Figure 3A, *S. pneumoniae* induced IL-8 and IL-6 release by BEAS-2B cells but at much lower levels than *P. gingivalis*. Much stronger IL-8 production and much weaker IL-6 production were observed with Detroit 562 cells (Figure 3B), whereas no significant cytokine release was observed with A549 cells (Figure 3C).

### 3.4. Involvement of TLR2 in P. gingivalis-Induced Proinflammatory Cytokine Production

**Figure 4:**
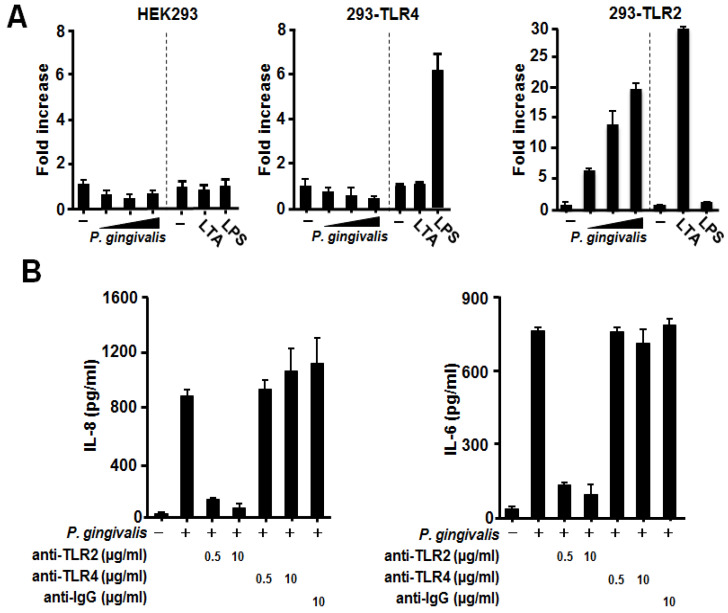

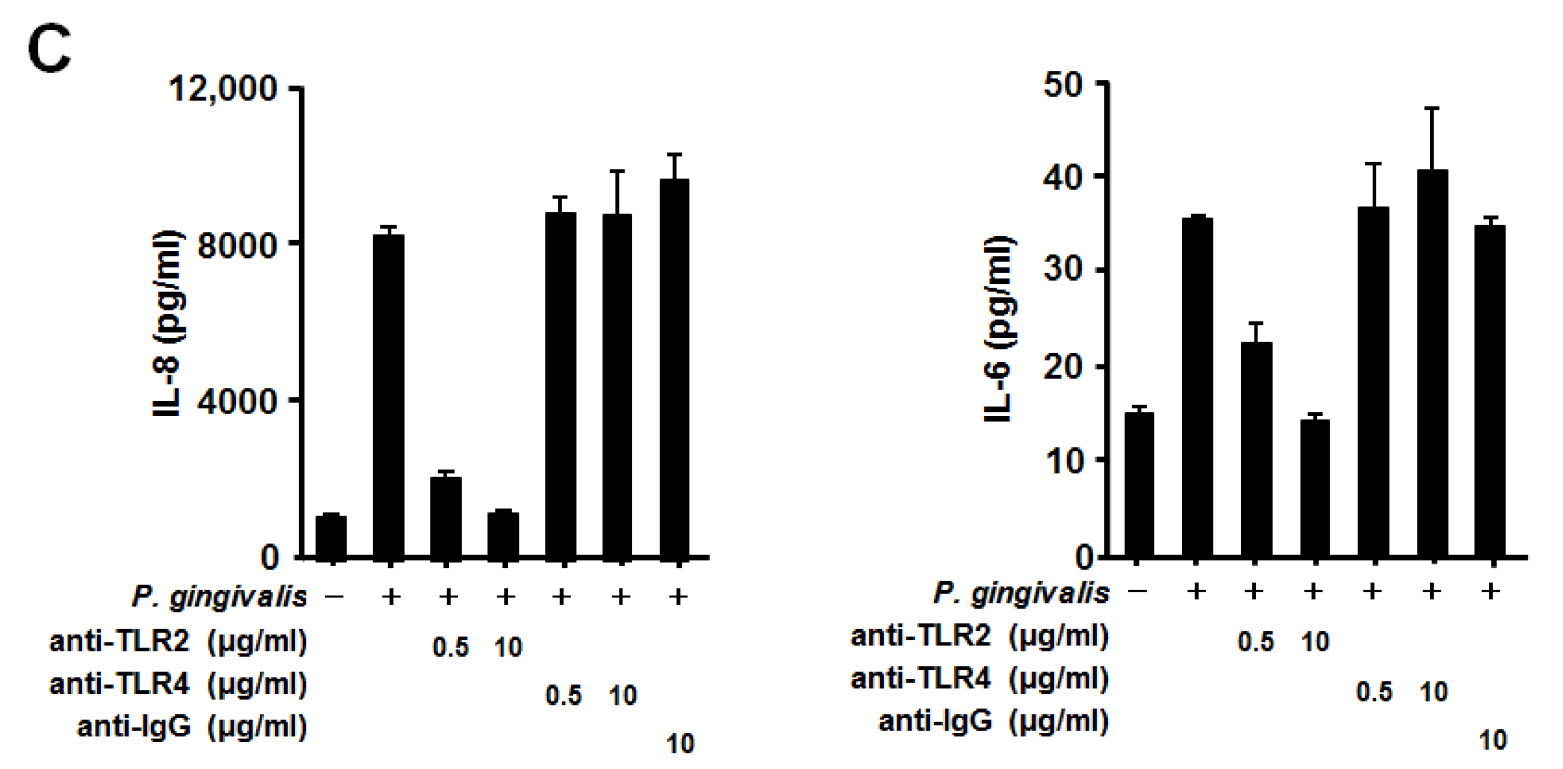
Involvement of Toll-like receptor (TLR)2 but not TLR4 in *Porphyromonas gingivalis*-induced proinflammatory cytokine production. (**A**) HEK293 cells, 293-TLR4 cells, or 293-TLR2 cells were transfected with a 5xκB-luc reporter plasmid together with an internal pRL-TK control plasmid. Twenty-four hours after transfection, these cells were stimulated with heat-inactivated *P. gingivalis* (1 × 10^7^, 0.5 × 10^8^, or 1 × 10^8^ CFU/mL), *S. aureus* LTA (5 μg/mL), or *E. coli* LPS (200 ng/mL) for 24 h. Luciferase activity in the whole cell lysate was then determined. The data are presented as the fold increase in luciferase activity relative to that in control transfected cells (no stimulation). Data are presented as the mean ± SD of three independent transfections.BEAS-2B (**B**) and Detroit 562 (**C**) cells were pretreated with the indicated concentrations of anti-TLR2, anti-TLR4, or control antibodies for 1 h and then stimulated with or without *P. gingivalis* (1 × 10^8^ CFU/mL). After 12 h incubation, culture supernatants were collected, and IL-8 and IL-6 levels were measured using enzyme-linked immunosorbent assay. These experiments were performed in triplicate, and data are presented as the mean ± SD.

Several studies have demonstrated TLR2 and TLR4 are important host receptors for recognizing *P. gingivalis* [10]. Eventually, these TLRs are reported to express on BASE-2B cells as well as on human bronchial epithelium and on Detroit 562 cells [23,24,25]. However, no study has yet examined whether human respiratory epithelial cells recognize *P. gingivalis* with their TLR2 or TLR4. In parallel, for producing IL-8 and IL-6, NF-κB is known to be an important transcription factor [26]. We therefore first examined whether these TLRs were involved in the NF-κB activation in *P. gingivalis*-stimulated epithelial cells using a luciferase assay. For this purpose, TLR-null HEK293 cells and HEK293 cells stably expressing either TLR2 or TLR4 cells were transfected with NF-κB reporter plasmids. As expected, NF-κB in 293-TLR2 cells were activated only by the stimulation with its specific ligand LTA from *S. aureus*, and likewise, 293-TLR4 cells were activated only by LPS from *E. coli*, whereas HEK293 cells were not activated by the both ligands (Figure 4A). When these cells were stimulated with *P. gingivalis*, 293-TLR2 cells alone were activated in a density-dependent manner (Figure 4A). These findings suggest that the observed cytokine production by BEAS-2B and Detroit 562 cells is induced via TLR2 but not via TLR4. To confirm this, BEAS-2B and Detroit 562 cells were treated with anti-TLR2 or anti-TLR4 antibodies prior to the stimulation with *P. gingivalis*. As shown in Figure 4B,C, anti-TLR4 antibodies had no effect on IL-8 and IL-6 production by either cell line following exposure to *P. gingivalis*, whereas the anti-TLR2 antibody significantly abrogated the production of both cytokines.

## 4. Discussion

Because bacteria in saliva are aspirated into the lower respiratory tract, many studies have revealed an association between chronic periodontitis and aspiration pneumonia. In fact, *P. gingivalis* has been isolated from BALF or sputum in patients with aspiration pneumonia [15,16,17,18]. However, the mechanism by which the bacterium triggers the development of aspiration pneumonia has not been delineated. By contrast, *S. pneumoniae* is a major exogenous respiratory pathogen that infects the bronchial epithelium. This subsequently induces the release of proinflammatory cytokines, thereby contributing to the development of pneumonia [2,3]. In the present study, we found that *P. gingivalis* is a significant inflammatory stimulant for bronchial and pharyngeal epithelial cells.

IL-8, a potent neutrophil chemoattractant and activator, is associated with the pathogenesis of pneumonia. It accumulates and subsequently degranulates neutrophils, resulting in the destruction of normal tissues [2,3]. In fact, IL-8 levels are markedly elevated in BALF or sputum from patients with pneumonia in relation to an increase of neutrophil counts [4,5,6]. Interestingly, the extent of IL-8 release by respiratory epithelia is positively correlated with the bacterial load in the lower respiratory tract, which consequently contributes to lung injury [27,28]. In this regard, secreted IL-6 is involved in the stimulation of acute-phase protein synthesis, leukocyte recruitment, B-cell differentiation, and T-cell activation in many chronic inflammatory diseases [2,3]. IL-6 levels are also increased in the plasma and BALF of patients with pneumonia [7]. We observed that the release of IL-8 and IL-6 by bronchial and pharyngeal epithelial cells, both of which were induced by the exposure by *P. gingivalis*, was stronger than that by *S. pneumoniae*. Because IL-8 and IL-6 exert their proinflammatory effects in paracrine and autocrine manners, they must elicit inflammatory responses in neighboring host cells. Our results thus suggest that inflammation in the lower respiratory epithelial cells is caused by an exposure to *P. gingivalis* itself and additionally by the absorption of paracrine proinflammatory cytokines released from the pharyngeal epithelium, which is induced by a precedent exposure to *P. gingivalis*. Therefore, these results may provide the novel insight that *P. gingivalis* contributes to the onset of aspiration pneumonia by inducing proinflammatory cytokine-production by bronchial and pharyngeal epithelial cells without infection.

From the aforementioned viewpoint, it should be also noted that elderly people have an increased risk of salivary aspiration because of their reduced laryngopharyngeal sensitivity and swallowing reflex impairment, both of which are ascribable to mild cognitive impairment [21]. In addition, an average person generates and ingests up to 1.5 L of saliva per day, which contains 1 × 10^8^/mL bacteria released from oral biofilms, indicating more than 1 × 10^11^ bacteria are swallowed daily [29,30]. Moreover, a high incidence of silent aspiration during sleep is eventually recorded in the elderly [31,32]. These observations along with our findings support our hypothesis that increased number of *P. gingivalis* in saliva because of poor oral hygiene raises the risk of the onset of aspiration pneumonia.

Respiratory epithelia represent the first line of defense against exogenous respiratory pathogens. Respiratory epithelia are surely exposed to aspirated *P. gingivalis*, after which the bacterium presumably attaches to epithelial surfaces via several interactions between epithelial receptors and bacterial ligands such as fimbriae and LPS [10]. However, recognition of bacterial attachment by the epithelium alone might induce cytokine production. The present findings of proinflammatory secretion by bronchial and pharyngeal epithelial cells exposed to heat-inactivated *P. gingivalis* indicate that infection is not necessarily required for the observed induction of cytokine production. In this connection, several studies have indicated that heat-inactivated *P. gingivalis* appears capable of modulating the expression of inflammatory cytokines in several types of human cells other than respiratory epithelial cells, such as monocytes, whole blood cells, and gingival epithelial cells [33,34,35,36]. These observations are reminiscent of the manner how *S. pneumoniae* induces pneumonia.

Significant involvement of TLR2 but not TLR4 of several types of human cells, such as periodontal ligament cells, gingival fibroblasts, and monocytes, gingival epithelial cells, in proinflammatory cytokine production induced by *P. gingivalis* is also reported [35,36,37]. Although both BEAS-2B and Detroit 562 cells express TLR2 and TLR4, to the best of our knowledge, we found for the first time that only anti-TLR2 antibody significantly and concentration-dependently reduced *P. gingivalis*-induced IL-8 and IL-6 production by these cells. In parallel with these findings, *P. gingivalis* can elicit high levels of proinflammatory cytokine production in wild-type or TLR4-deficient mice, but not TLR2-deficient mice [38,39]. This suggests that without heat inactivation, TLR2 is necessary for *P. gingivalis* to induce proinflammatory cytokine production by host cells. These observations along with our findings suggest that bronchial and pharyngeal epithelia secrete some proinflammatory cytokines via TLR2 stimulation and become inflamed quickly when they are exposed to *P. gingivalis* irrespective of bacterial vitality.

Although *P. gingivalis* LPS and Fim A, a major fimbriae of *P. gingivalis*, have been widely accepted as TLR2 ligands [40,41,42], the stimulation of neither BEAS-2B nor Detroit 562 cells by these bacterial surface structures alone elicited IL-8 and IL-6 protein secretion. Therefore, the necessity of other surface structures or the whole *P. gingivalis* cell for the observed cytokine production is currently under study. In addition, in the human phagocytes, TLR2 is an essential molecule for sensing *P. gingivalis* and appears to be paired often by TLR1 and sometimes by TLR6 for arranging subsequent intracellular signaling due to imminent immunological necessity [40,43]. Because this has not been investigated in human respiratory epithelia, it would be interesting to see how blocking of TLR1 or TLR6 influences the *P. gingivalis*-induced IL-8 and IL-6 production. Further studies are therefore needed. However, it would be important that our findings have revealed a putatively causal relationship between *P. gingivalis* as a proinflammatory stimulant to several human respiratory epithelia and the onset of aspiration pneumonia.
